# Combined AOPs for Formaldehyde Degradation Using Heterogeneous Nanostructured Catalysts

**DOI:** 10.3390/nano10010148

**Published:** 2020-01-14

**Authors:** Renato Bonora, Carlo Boaretti, Laura Campea, Martina Roso, Alessandro Martucci, Michele Modesti, Alessandra Lorenzetti

**Affiliations:** Department of Industrial Engineering, University of Padova, Via Marzolo 9, 35131 Padova, Italy; renato.bonora@unipd.it (R.B.); lcampea94@gmail.com (L.C.); martina.roso@unipd.it (M.R.); alex.martucci@unipd.it (A.M.); michele.modesti@unipd.it (M.M.)

**Keywords:** formaldehyde, titanium dioxide, photocatalysis, photo-Fenton, heterogeneous catalyst, combination, synergy, AOPs, nanofibers

## Abstract

In this paper we studied the combination of advanced oxidation processes (AOPs), i.e., TiO_2_-based photocatalysis and photo-Fenton process, on the degradation of aqueous solutions containing a low (90 ppm) concentration of formaldehyde. Heterogeneous nanostructured catalysts, supported on polymeric nanofibers, were used; for comparison, some homogeneous or partly heterogeneous systems were also analyzed. Furthermore, to make the process more sustainable (in terms of costs and safety) no hydrogen peroxide was added to the system. The results showed that the combination of AOPs gave a synergy since the presence of iron was beneficial in promoting the photocatalytic activity of TiO_2_ while TiO_2_ was beneficial in promoting the photo-Fenton reaction. Moreover, very good results were obtained using fully heterogeneous nanostructured catalysts (based on TiO_2_ and FeSO_4_), without the need to add H_2_O_2_.

## 1. Introduction

Terrorism is a threat that, in recent decades, has become increasingly fearsome and destructive. It is known that chemical weapons and toxic compounds are used by terrorists in their attacks. For this reason, toxic industrial materials (TIMs), which are commonly used in the chemical industry, are the subject of discussion in many countries, where governments want to analyze and optimize the conditions of use and disposal of these hazardous chemicals. TIMs are characterized by high toxicity and wide availability [1]. This problem has led to the introduction of restrictive regulations on trade of these materials and on safety in chemical industries which, having a large quantity of toxic materials, are a sensitive target for possible terrorist actions and, therefore, require an adequate TIMs degradation system. To limit the spread of these substances, wastewater from industries, which could contain toxic compounds in high concentrations, should also be treated. One of the most dangerous TIMs is formaldehyde, a suspected carcinogenic chemical compound characterized by high toxicity, which is widely used in various sectors of the chemical industry (synthetic resins, insulators, paints, textiles) [2].

Advanced oxidation processes (AOPs) are considered the most effective treatments for the removal of these species from water [3], because they allow a complete mineralization even of the most refractory organic compounds, thus destroying wastewater pollutants and transforming them into less and even non-toxic products, thereby providing an ultimate solution for wastewater treatment.

Among the most widespread radical species used in AOPs, hydroxyl radical plays a primary role, since it is the most reactive oxidizing agent in water treatment (having a high oxidizing power [3]), it is very non-selective in its behavior and rapidly reacts with numerous species. Because hydroxyl radicals have a very short lifetime, they are only in situ produced during application through different methods, including a combination of oxidizing agents (e.g., H_2_O_2_), irradiation (such as ultraviolet (UV) light, or ultrasound), and catalysts (such as Fenton reagent, Fe^2+^, or titanium dioxide, TiO_2_) [4]. Indeed, hydroxyl radicals can be initiated by photons in the presence of catalysts (the most common being titanium dioxide, TiO_2_) or oxidants such as H_2_O_2_ (a H_2_O_2_ molecule is cleaved by UV irradiation to generate two OH•); they are also produced in the presence of some metals (e.g., iron) which are able to activate H_2_O_2_. For example, in the so-called Fenton process, H_2_O_2_ reacts with Fe^2+^ to generate strong reactive species, like hydroxyl radicals, although other substances such as ferryl ions are proposed [5].

Several papers have already reported on the degradation of aqueous formaldehyde solution by photocatalytic process using titanium dioxide [6,7,8,9]. Some of them have used homogeneous catalysis, i.e., TiO_2_ particles suspended in contaminated water, which makes it necessary to recover them after treatment while the heterogeneous (i.e., supported) catalyst configurations, on the other hand, eliminate the need for catalyst filtration but generally result in a significant reduction in system efficiency [10].

Fenton and photo-Fenton processes have already been proposed for formaldehyde removal from wastewater [11,12,13]. In general, the Fenton and photo-Fenton processes require the use of ferrous ions (Fe^2+^) and hydrogen peroxide (H_2_O_2_) [14]. Thus, large amounts of H_2_O_2_ need to be irreversibly consumed in real application to treat wastewater containing a high concentration of organic pollutants, and considerable iron dosage is indispensable if higher efficiency is required [15,16]. In the Fenton process, there is a need for continuous addition of ferrous ion into the reaction medium for the reaction to proceed further. This disadvantage could be overcome by the use of photo-Fenton reagent, which is a cyclic process and regenerates Fe^2+^ ion [15], thus limiting the deposition of ferric ion sludge.

Based on the similarity between the mechanism of destruction in the case of different advanced oxidation processes (AOPs) [17], some research efforts focusing on the synergism between several AOPs have already been published. Mokhbi et al. [18] studied the effectiveness of photocatalysis with Fenton’s reagent for treating oily wastewater; Kim et al. [19] studied the combined photocatalytic- and photo-Fenton system for treating some organic substrates (i.e., phenol, benzoic acid, and methanol); Xu et al. [15] studied interactions between TiO_2_ and Fe^3+^/H_2_O_2_ to degrade bisphenol A. They all used unsupported catalysts, i.e., TiO_2_ and Fe^2+^ were put in solution, in the presence of H_2_O_2_ and acidic pH, and found that synergistic effects resulted. In other works, the combination of TiO_2_ and Fe ions has been proposed but without exploiting Fenton or photo-Fenton reactions; the main aim of those works was, indeed, to dope titania in order to modify its photocatalytic features, such as visible light absorption and photocatalytic efficiency. For example, Khanmohammadi et al. [20] studied the use of unsupported catalyst, composed of sol-gel synthesized Fe_2_O_3_-TiO_2_ nano hybrid, in the degradation of liquid-phase formaldehyde in the presence of ultraviolet and visible irradiation. They found that the degradation took place according to a two-step mechanism: first, formaldehyde was converted to formic acid and then this was converted to H_2_O and CO_2_ during a photocatalytic reaction via radical mechanism in the presence of UV irradiation. In a similar way, Siddhapara et al. [21] used sol-gel TiO_2_ nanoparticles doped with a transition metal (Mn, Fe, Co) to degrade liquid formaldehyde solution; they found that the substitution with transition metals enhanced photocatalytic efficiency since transition metals increased defect sites and acted as a permanent space charge region, whose electric force improved the separating efficiency of electron-holes. Li et al. [22] studied the effect of the content of Fe on the photocatalytic performance of doped TiO_2_ on the degradation of gaseous formaldehyde and found that doped TiO_2_ exhibited higher photocatalytic activity under visible light irradiation. Cheng et al. [23] mixed TiO_2_ with iron oxide to produce modified photocatalysts (Fe/TiO_2_) and then coated them on a supporting medium of fiberglass and studied the degradation of gaseous formaldehyde using these supported catalysts. They concluded that the enhancement of formaldehyde decomposition by iron-doped TiO_2_ may be attributed to the facts that the doped iron ions could effectively enhance the absorption of UV-visible wavelength, increase the photocatalytic reactivity and retard the recombination of electron and electron-hole pairs, which thus contributed to the increase of formaldehyde decomposition efficiency. Chun et al. [24] studied the photocatalytic degradation of gaseous pollutants (benzene, toluene, ethyl benzene, and o-xylene (BTEX)) by preparing the Fe-TiO_2_ catalyst by the sol-gel technique and electrospinning it in presence of polyvinyl pyrrolidone; they found enhanced photocatalytic activity in presence of optimal Fe:TiO_2_ ratio.

However, authors using Fe-doped titania [20,21,22,23,24] did not mention the photo-Fenton reaction and did not adjust pH accordingly.

Nevertheless, in literature there are already some examples of combination of photocatalysis and photo-Fenton using supported catalysts. For example, Bouras et al. [25] combined a titania-supported photocatalyst as well as Photo-Fenton oxidation, by using FeCl_2_⋅4H_2_O in solution, for the decolorization of aqueous solution of the azo dye Basic Blue 41. Only very few examples using a fully supported catalytic system based on TiO_2_ and Fe ions and exploiting photo-Fenton reaction can be found. For example, Quici et al., degraded oxalic acid [26], while Shao et al. [27] used TiO_2_ and Fe(III) *meso*-tetraphenylporphyrin on polystyrene nanofibers to degrade methyl orange under visible light. They electrospun solutions simultaneously containing polystyrene, porphyrin and TiO_2_ and found that this system, in presence of added H_2_O_2_, coupling photocatalysis and Fenton reaction gave the best performance. Zhang et al. [28] used TiO_2_/Cu_2_O composite coated on glass matrix to degrade methylene blue using both photocatalysis and the Fenton process, using in situ generated H_2_O_2_. In the paper of Zhu et al. [29], beta-iron oxyhydroxide (β-FeOOH) nanostructures on electrospun TiO_2_ nanofibers were synthesized and used to degrade methyl orange. They showed that the degradation was faster in the presence of added H_2_O_2_ but it still took place also without adding it.

In this paper we study the combination of TiO_2_-based photocatalysis and photo-Fenton process on the degradation of aqueous solutions containing a low (90 ppm) concentration of formaldehyde to understand if synergy can be revealed. Heterogeneous nanostructured catalysts, supported on polymeric nanofibers, have been used; for comparison, some homogeneous or partly heterogeneous systems have also been analyzed. Furthermore, to make the process more sustainable (in terms of costs and safety) no hydrogen peroxide has been added to the system. To the best of our knowledge, this combination of advanced oxidation processes has never been studied in the literature for formaldehyde degradation.

## 2. Materials and Methods

### 2.1. Materials

Polyacrylonitrile (PAN, Mw = 150,000 g/mol, Sigma Aldrich, St. Louis, MO, USA) was used as support for the catalytic systems in electrospun form; *N*,*N*-dimethylformamide (DMF, Sigma-Aldrich, St. Louis, MO, USA) was used as electrospinning solvent. Ferrous sulfate (FeSO_4_⋅7H_2_O) was used as a catalyst for the photo-Fenton process and was supplied by Sigma-Aldrich (St. Louis, MO, USA). A crystalline, titanium dioxide powder was used for photocatalytic process, i.e., Aeroxide^®^ TiO_2_ P90 (d = 14 nm, BET = 90 m^2^/g, 90/10 anatase/rutile, crystal size = 11 nm [30]), supplied by Evonik (Essen, Germany). We decided to use this one (instead of the most commonly used P25) because some preliminary trials (unpublished results) have shown that P90 was more active than P25 in our degradation systems. Some other authors reported similar results for different pollutants when using supported TiO_2_-based catalysts [30,31].

The aqueous formaldehyde solution for testing the catalytic activity was prepared by diluting formalin (37% w/w of formaldehyde and 10% w/w of methanol), supplied by Scharlau (Barcelona, Spain), with distilled water to reach a final formaldehyde concentration of 90 ppm. When the photo-Fenton process was considered, it was necessary to use 0.2 N sulfuric acid (H_2_SO_4_ at 98% w/w, Sigma Aldrich, St. Louis, MO, USA) to set the pH around 3, because this type of process is favored by acid pH [12]. Hydrogen peroxide was used as was a 30% (w/w) solution, supplied by Sigma Aldrich (St. Louis, MO, USA).

Finally, pentafluorobenzylhydroxylamine hydrochlorinated (PFBHA-HCl), purchased by Alfa Aesar (Haverhill, MA, USA), and 1,2-dibromopropane 98% w/w as internal standard (Sigma-Aldrich, St. Louis, MO, USA) dissolved into hexane (Sigma-Aldrich, St. Louis, MO, USA) have been used for the derivatization and extraction process, needed to quantify formaldehyde content in aqueous solutions by gas chromatography-mass spectrometry (GC-MS), according to the U.S. Environmental Protection Agency (EPA) method 556.

### 2.2. Preparation of the Nanostructured Catalytic Systems

**Figure 1:**
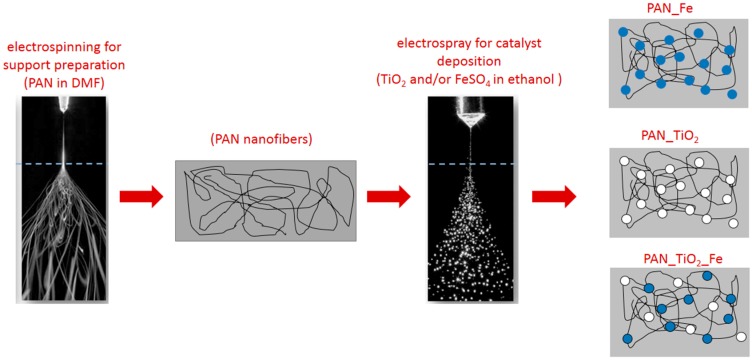
Schematic procedure for the preparation of nanostructured catalytic systems.

Supported nanostructured catalysts have been used. The support was made by electrospun PAN nanofibers, while electrospraying was used for the catalysts’ deposition. In this way, the catalysts were completely placed on the upper surface of the membrane and so they were homogeneously irradiated by ultraviolet (UV) rays [32]. A schematic procedure is depicted in Figure 1. The use of nanofibers membranes as support allowed to have a very large surface area where the catalysts can be deposited.

The first step in preparation was the production of PAN nanofiber membranes by electrospinning, using a solution of PAN in DMF at 5% w/w, which must be stirred for not less than 15 h. The homogeneous mixture was then transferred into a 5 mL syringe fitted with a 27 G gauge needle. The syringe was put horizontally on a pump (NE-300 single syringe pump, NewEra Pump System Inc., Farmingdale, NY, USA) and the positive electrode of the high voltage power supply (Gamma High Voltage Inc., Ormond Beach, FL, USA), capable of generating voltages up to 60 kV, was clamped to the metal needle tip. The nanofibers were deposited on a metallic rotating collector. The process parameters are reported in Table 1.

For the catalysts deposition by electrospraying process, the catalysts were suspended into ethanol. TiO_2_ was used at 5% w/w; this concentration was chosen in order to obtain a catalyst content of about 0.14 mg/cm^2^, which was, based on our previous experience [32], the optimal one to have a widespread distribution of titanium dioxide, avoiding great clusters, agglomeration and clogging of the nanostructured substrate. Moreover, at higher concentrations, an increase in opacity and light scattering of TiO_2_ particles occurs, leading to a decreased passage of irradiation through the sample [10]. For ferrous sulfate, based on mass balance calculation, a 4% w/w solution in ethanol should be enough to have the same catalyst content of titania and similar to that used in literature for a homogenous photo-Fenton system [11]. However, due to a partial loss of FeSO_4_ experienced during the electrospray process, owing probably to an interaction of the Fe^2+^ with the electric field, a 10% w/w FeSO_4_ solution in ethanol has to be used. When the two catalysts were employed together, TiO_2_ was used at 5% w/w and the FeSO_4_ at 10% w/w dispersion in ethanol, in order to deposit a 50:50 TiO_2_:FeSO_4_ catalytic system on to the PAN support.

The catalysts’ dispersions (TiO_2_ and/or FeSO_4_ in ethanol) were sonicated for 40 min at 40% of amplitude and constant temperature by using ultrasonic probe VC505^®^ of Sonics & Materials (power of 500 W and a probe length of 254 mm, Newtown, CT, USA). Then, Dynasylan^®^ 4144 was added at 1% w/w of ethanol, followed by a further sonication (15 min).

Similarly, to the electrospinning, the catalysts’ dispersion was put in a syringe of 5 mL volume and then it was electrosprayed onto the preformed electrospun PAN membrane thanks to a voltage generated between the metallic needle and the collector. The whole production process was carried out at room temperature and the humidity was kept constant by flowing dried compressed air.

In the following, membranes are named PAN_TiO_2_, PAN_Fe and PAN_TiO_2__Fe if the catalyst deposited was TiO_2_, FeSO_4_ or TiO_2_+FeSO_4_, respectively. In this work, we referred to homogeneous process when the catalyst (Fe^2+^) and/or oxidant (H_2_O_2_) were in the same (liquid) phase of formaldehyde (i.e., they are simply added to formaldehyde solution) while heterogeneous one referred to the use of supported TiO_2_ and/or Fe catalyst prepared by electro-spinning and -spraying.

Concentrations of catalysts, in both homogeneous and heterogeneous experiments, have been fixed based on optimal compositions reported in literature [11] or based on our previous experience [32]: for homogeneous catalysis, FeSO_4_ and H_2_O_2_ were used at 24 and 400 ppm, respectively, while in the heterogeneous supported system around 0.15 mg/cm^2^ of catalyst has been deposited (the exact values are obtained by thermogravimetric analysis (TGA) and are reported in the following).

### 2.3. Testing of the Catalytic Systems in the Degradation of Aqueous Formaldehyde Solution

**Figure 2:**
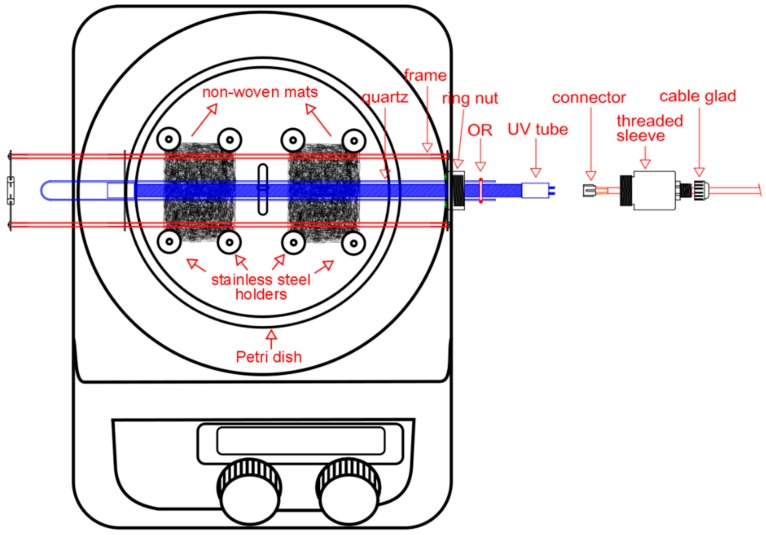
Experimental set-up for testing catalytic activity in formaldehyde degradation.

The set-up used for the experiments is shown in Figure 2. It consisted mainly of a Petri dish with a diameter of 15 cm and a height of 2 cm, in which 70 mL of aqueous formaldehyde solution (90 ppm) is placed; a magnetic stirrer provided a good mixing inside the system. The catalytic membrane, comprising titanium dioxide and/or ferrous sulfate, with a 50 cm^2^ area, was placed on the bottom of the Petri dish and it was kept fixed using eight stainless steel weights. UV irradiation is provided by a lamp (16 W UV Stylo E16, Light Progress, Anghiari, Italy) with a spectral peak centered around 250 nm. The distance between the lamp and the membrane was constant (2 cm). For some trials, ferrous sulfate was added directly to the aqueous solution. In this case the experiment started after the complete solubilization of the salt into the solution.

The catalytic process lasted 4 h. During the tests, some samples were taken from the aqueous solution. These samples were subjected to a derivatization process according to EPA method 556, before being injected into a GC-MS to quantify formaldehyde content.

It is known that bubbling air within the formaldehyde solution gives some benefits in terms of degradation rate [6] through the electron transfer reaction generating superoxide radical anion:

$$e^{-}+O_{2}\;\to \;O_{2}^{-}•$$

However, we preferred to avoid the use of air in order to avoid misleading results due to formaldehyde stripping by the bubbling air. Nonetheless, due to traces of (electron acceptor) impurities or adventitious O_2_ in the solution (since the solution is in direct contact with ambient air) some superoxide radical anions can still form [6].

### 2.4. Characterization

All the membranes are characterized in terms of morphology by scanning electron microscopy (SEM, Obducat CamScan MX2500, Cambridge, UK) and transmission electron microscopy (TEM, FEI model Tecnai G12, Hillsboro, OR, USA). Sample preparation for TEM imaging have been carried out as follows: the membrane samples were embedded in a proper epoxy resin and slices of 100 nm thickness were obtained by ultramicrotomy of the cured resin.

Furthermore, catalytic content deposited onto the PAN support was investigated through TGA (SDT Q600, TA Instruments, New Castle, DE, USA); the analysis was performed using alumina pan with air flow of 100 cc/min and a heating rate of 20 °C/min, from room temperature to 1000 °C.

The quantification of formaldehyde was carried out by withdrawing samples from the aqueous solution during the degradation tests and derivatizing them using o-(2,3,4,5,6-Pentafluorobenzyl)hydroxylamine hydrochloride (PFBHA-HCl). The oxime derivative formed was extracted from water with 4 mL hexane. The extract was processed through an acidic wash step and then analyzed by GC-MS (Gas Chromatograph Trace 1300^®^ coupled with the Single Quadrupole Mass Spectrometer ISQ QD^®^, both purchased from Thermo Scientific, Waltham, MA, USA). The GC column was a non-polar DB5 capillary column (0.25 mm i.d., 30 m length, supplied by Agilent, Santa Clara, CA, USA). According to the EPA method 556, an internal standard (1,2-dibromopropane) has been used to obtain quantitative data. A calibration curve has also been built before starting the experimental tests, by analyzing formaldehyde solutions of known concentration. The target analytes (oxime derivative, to quantify formaldehyde, and internal standard) were identified (m/z 225 and 121, respectively) and quantified by using a calibration curve. Each data point has been obtained as the mean value of three replicates.

## 3. Results and Discussion

### 3.1. Catalytic Membranes Characterization

**Figure 3:**
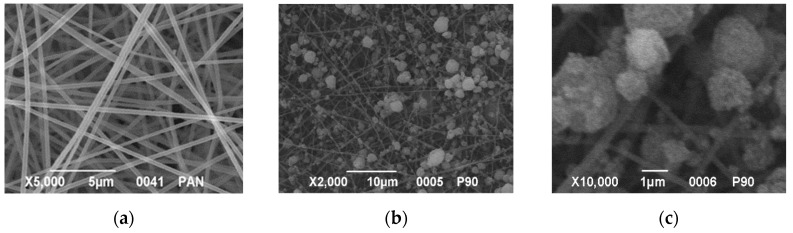
Scanning electron microscope (SEM) micrographs of (**a**) PAN, (**b**,**c**) PAN_TiO_2_ membranes.

All the produced membranes were characterized by SEM analysis in order to gain a better understanding of the morphology. The SEM micrographs of the PAN and PAN_TiO_2_ membranes are reported in Figure 3. It can be observed that PAN nanofibers were smooth, with a mean diameter of ~300 nm. The TiO_2_ nanoparticles had the tendency to create clusters when spread over the nanofibers surface and some large aggregates were formed. However, the catalytic particles had an almost homogeneous distribution upon PAN support without clogging it.

**Figure 4:**
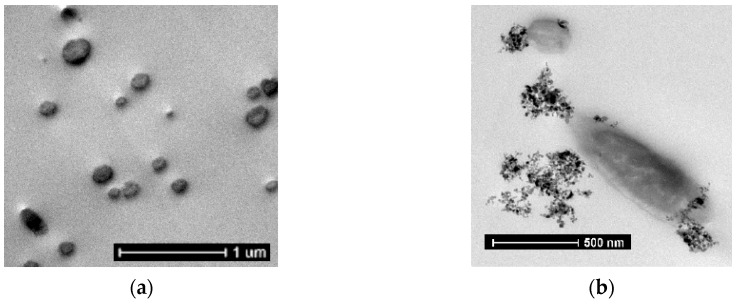
Transmission electron microscope (TEM) images at different magnifications (**a**,**b**) of PAN_TiO_2_ membrane.

**Figure 5:**
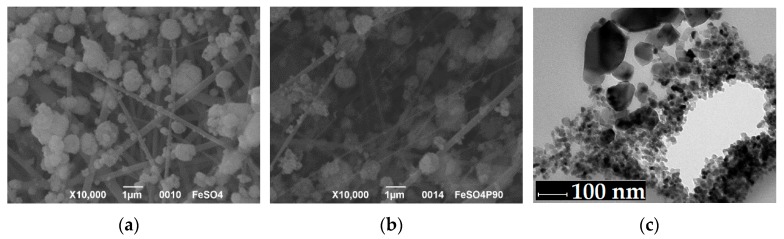
SEM images of (**a**) PAN_Fe and (**b**) PAN_TiO_2__Fe membranes; (**c**) TEM image of PAN_TiO_2__Fe membrane.

TEM images of PAN-TiO_2_ are reported in Figure 4. In Figure 4a, the cross sections of the nanofibers can be observed; in Figure 4b, TiO_2_ nanoparticles can be seen around the nanofibers (the cross section of the fiber is not circular due to cutting direction); TiO_2_ clusters were, mainly, less than 500 nm. From SEM and TEM analysis of PAN_Fe membranes (Figure 5), it was evident that this kind of catalyst also covered almost completely the nanofibers of PAN; in contrast to what happens for TiO_2_, the clusters had larger dimension and no spherical shape. The difference between the two catalysts is more clear if the TEM image is analysed: ferrous sulfate had bigger dimensions and irregular shape, while TiO_2_ appeared in spherical clusters with submicron mean diameter.

The catalyst content of each prepared membrane was measured by TGA analysis, analyzing the residue at high temperature (1000 °C), taking into account that TiO_2_ did not lose any weight at that temperature while FeSO_4_ lost 70% of its initial weight. Based on TGA results, the specific catalyst content for each membrane has been measured; the results are reported in Table 2.

### 3.2. Photocatalytic Degradation of the Formaldehyde

**Scheme 1:**
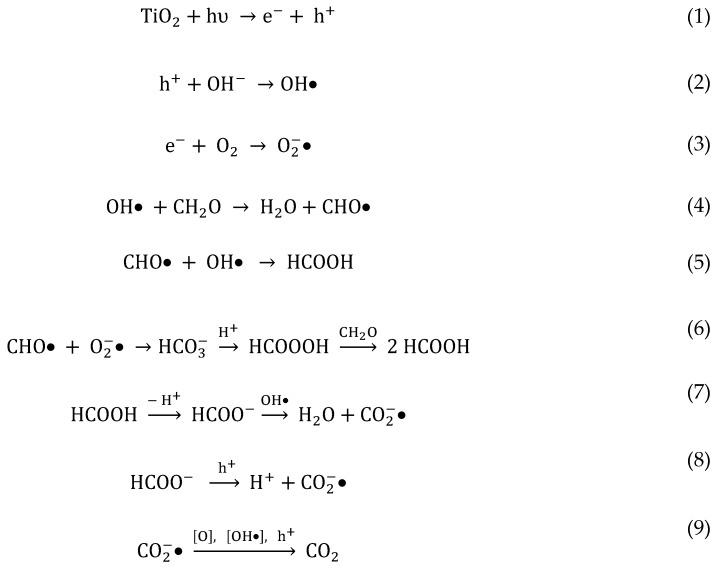
Mechanism of photocatalytic degradation of formaldehyde in aqueous solution.

**Figure 6:**
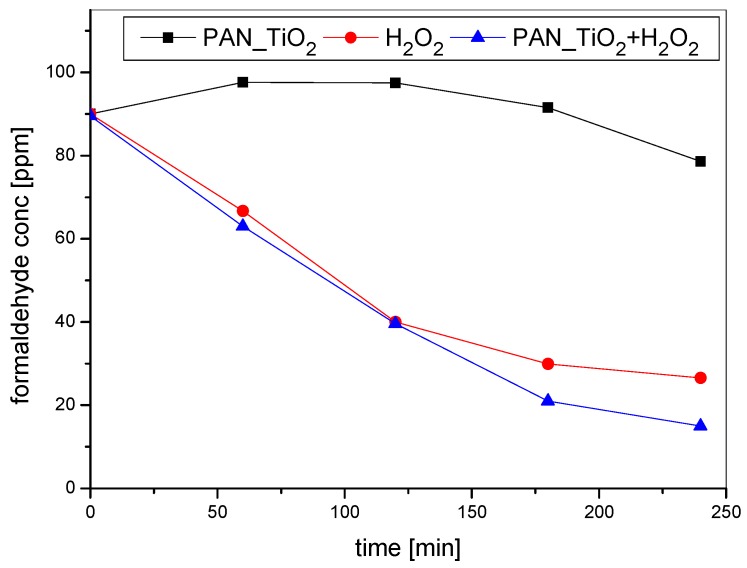
Formaldehyde concentration during photocatalytic experiment with and without added H_2_O_2_ (400 ppm). The maximum standard deviation of the experimental points was 1.9 ppm.

The photocatalytic degradation of formaldehyde, in the presence of TiO_2_, takes place according to the following reactions (Scheme 1, [33]):

The results for catalytic degradation with PAN_TiO_2_ membrane are reported in Figure 6; PAN_TiO_2_ membrane contained 0.14 mg/cm^2^ of TiO_2_ (i.e., optimal composition defined elsewhere [32]) which corresponded to a total amount of TiO_2_ of 7 mg in the system.

**Scheme 2:**
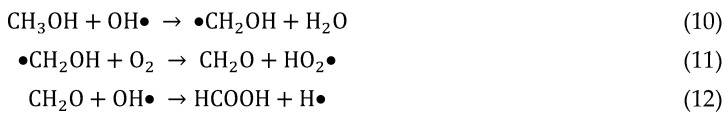
Methanol photocatalytic degradation path.

For heterogeneous catalytic system PAN_TiO_2_, an increase in the formaldehyde content was observed during the first stage of degradation. This can be rationalized considering that the degradation of methanol (which was contained at 10 wt% in the formalin solution as stabilizer to limit formaldehyde polymerization) led to the formation of formaldehyde, according to the following reactions (Scheme 2, [34]):

The degradation of the methanol can start as soon as a hydroxyl radical is produced and it may compete with formaldehyde to consume hydroxyl radicals resulting in the retardation of the oxidation reaction of formaldehyde. Both methanol and formaldehyde formed formic acid during their degradation. Indeed, the initial pH of the solution was around 6.5–7, and it was not adjusted, owing to the pH-independent photocatalytic activity of TiO_2_ [19], but during the test, it decreased to 4.2 and then rose to 5, as already reported in similar studies [6,7], thus implying the formation of formic acid. Therefore, we can assess that, since both methanol and formaldehyde were oxidized to formic acid (and then to CO_2_ and water), these compounds were responsible for the drop in the pH of the solution. As CO_2_ was liberated from the solution, pH increased [7].

However, it was evident that after 4 h the abatement of the formaldehyde content was still low.

The addition of H_2_O_2_ (400 ppm, according to optimal content reported in [11]) in aqueous solution to the PAN-TiO_2_ system (PAN_TiO_2_ + H_2_O_2_ in Figure 6) resulted in greater oxidation with respect to neat PAN-TiO_2_ and this was likely attributed to the enhanced charge separation induced by H_2_O_2_ as an electron acceptor and the reductive conversion of H_2_O_2_ to OH• [7]. Moreover, the methanol degradation was very fast and the increase in the formaldehyde content was not revealed (since the first sampling point was after 1 h).

Comparing homogeneous system using H_2_O_2_ only (400 ppm, without catalytic membrane, curve H_2_O_2_ in Figure 6) with the previous heterogeneous one (PAN_TiO_2_ + H_2_O_2_), it can be noted that the higher removal rate was mainly due, especially in the first 2 h, to the direct oxidation of H_2_O_2_ onto the organic substrate. The trend of the degradation of formaldehyde with only H_2_O_2_ was very similar to that reported by Guimarães et al. [7]. Since also in H_2_O_2_-system the pH decreased from 6.5 to 3.8, the formation of formic acid can still be speculated. After the H_2_O_2_ was completely consumed, the degradation reaction stopped and the CH_2_O concentration reached a plateau while in the presence of TiO_2_ it seemed to further decrease.

### 3.3. Photo-Fenton Degradation of Formaldehyde

Photo-Fenton degradation of formaldehyde was also studied. Several catalytic, homogeneous and heterogeneous systems have been compared, to gain deep understanding of the process. In this work, we referred to homogeneous process when the catalyst (Fe^2+^) and/or oxidant (H_2_O_2_) were in the same (liquid) phase of formaldehyde while the heterogeneous one referred to the use of supported Fe catalyst.

**Scheme 3:**
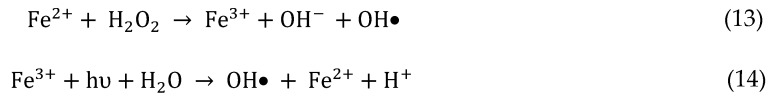
Photo-Fenton reactions.

Generally speaking, photo-Fenton systems are based on the contemporary use of Fe ions and hydrogen peroxide: ferrous ions react with hydrogen peroxide under the irradiation of UV light, producing hydroxyl radicals with powerful oxidizing abilities. While in the Fenton reaction Fe^3+^ ions accumulate in the system and the reaction stops once all Fe^2+^ ions are consumed, in the photo-Fenton reaction the regeneration of ferrous ions by photo-reduction of ferric ions occurs according to Scheme 3:

The newly generated ferrous ions react again with H_2_O_2_ generating hydroxyl radical and ferric ion and, in this way, the cycle continues. Therefore, the rate of degradation of organic pollutants in photo-Fenton reaction increases with respect to the Fenton one [35]. The photo-Fenton process offers better performance at pH 3.0, when the hydroxyl-Fe^3+^ complexes are more soluble and Fe(OH)^2+^ is more photoactive [36]. However, although ferrous ions are regenerated, generally, a large amount of H_2_O_2_ needs to be irreversibly consumed.

**Figure 7:**
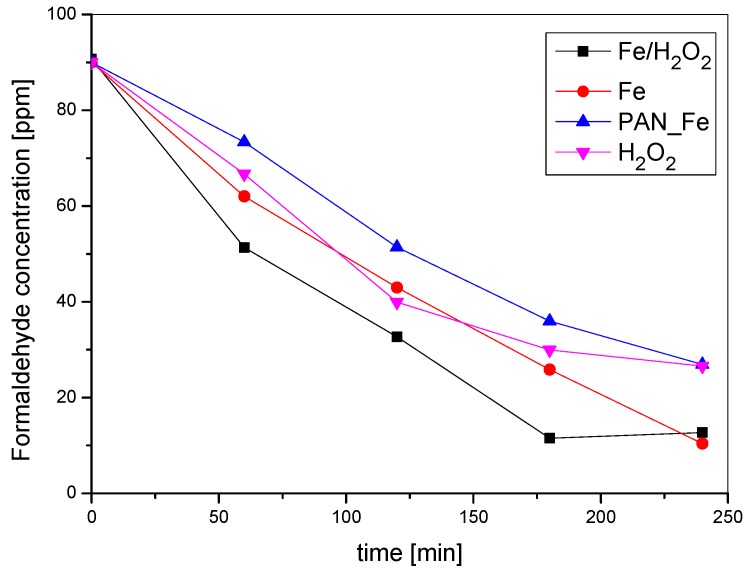
Formaldehyde concentration during photolysis and photo-Fenton experiment. The maximum standard deviation of the experimental points was 2.5 ppm.

Results for formaldehyde degradation in a homogeneous photo-Fenton process, with and without added H_2_O_2_ (Fe/H_2_O_2_ and Fe curves, respectively) as well as heterogeneous one (PAN_Fe curve) are reported in Figure 7; homogeneous photolytic oxidation (H_2_O_2_ curve in Figure 7) is also reported for comparison. In photo-Fenton processes (i.e., whenever Fe was present) the pH has been adjusted to 3 using 0.2 N sulfuric acid. When ferrous sulfate and H_2_O_2_ were used in solution, their concentrations were 24 and 400 ppm, respectively, i.e., set at the optimal conditions identified elsewhere [11]. The heterogeneous PAN-Fe catalyst comprised 0.12 mg/cm^2^ of FeSO_4_ (i.e., similar content to the TiO_2_ content of the previous experiment, i.e., optimal composition defined elsewhere [32]), which corresponded to a total of 6 mg of ferrous sulfate in the system. All the trials have been carried out under UV irradiation; it shall be kept in mind that in presence of iron sulfate the UV absorbance of the system around 290 nm was quite high, owing to the absorption properties of this compound [23].

Within the homogeneous processes (curves Fe, H_2_O_2_ and Fe/H_2_O_2_ in Figure 7), the photo-Fenton Fe/H_2_O_2_ demonstrated the best performance; however, also the use of iron compound alone (Fe curve), without adding H_2_O_2_, gave good results since the final formaldehyde concentration was the same as for Fe/H_2_O_2_, although the initial rate was lower. Similar results have already been reported by other authors [29,37] for different compounds, i.e., the addition of H_2_O_2_ to Fe catalyst was beneficial, but the degradation took place also without adding H_2_O_2_.

**Scheme 4:**
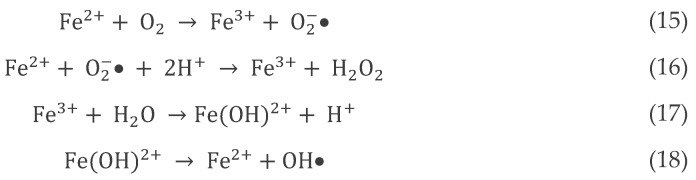
Reactions between ferrous ion and dissolved oxygen.

There can be several reasons for this. First, it should also be kept in mind that small amount of H_2_O_2_ can also be developed during UV irradiation of formaldehyde [9]. Secondly, ferrous ion can react with dissolved oxygen in water (Scheme 4) forming reactive oxidants capable of oxidative degradation. Indeed, superoxide radical anions can form, by reaction of Fe^2+^ with oxygen of the air dissolved into the solution [6], leading to the formation of H_2_O_2_ [38]; moreover, Fe^2+^ in the UV light conditions, can be partially transformed into Fe^3+^, the Fe^3+^ in solution can be hydrolyzed to hydroxylable Fe(OH)^2+^, Fe (OH)^2+^ also can be transformed into Fe^2+^ under ultraviolet light, while producing OH radicals to form a Fe^3+^/Fe^2+^ cycle of reactions [11,39,40]:

Therefore, in the presence of Fe^2+^ and UV light several reactions can take place to generate oxidizing species (OH•, $O_{2}^{-}•$ and H_2_O_2_) without the need to add external hydrogen peroxide.

Lastly, it could also be possible that the formic acid, deriving from degradation of formaldehyde, created a complex compound with iron [41] which could be photolyzed and even photocatalyzed in the reaction medium, generating Fe^2+^, H_2_O_2_ and other active radical species [38], in a similar way to that reported for oxalic acid by Quici et al. [26].

The effectiveness of Fe catalyst also without H_2_O_2_ is very important since avoiding the use of H_2_O_2_ makes the process more economic and safer.

Also, a heterogeneous photo-Fenton process, i.e., using a supported Fe catalyst (PAN_Fe), has been shown to be active in formaldehyde degradation; however, as expected, the reaction rate was slower with respect to homogenous systems. This PAN_Fe system reached the same final formaldehyde content as photolytic oxidation (H_2_O_2_ curve) process (but without the use of costly and hazardous reagents like water peroxide) and only slightly higher concentration than homogenous photo-Fenton processes.

### 3.4. Photo-Catalytic-Fenton Degradation of Formaldehyde

After the separate analysis of photocatalytic and photo-Fenton processes, the combination of such AOPs has been studied.

**Figure 8:**
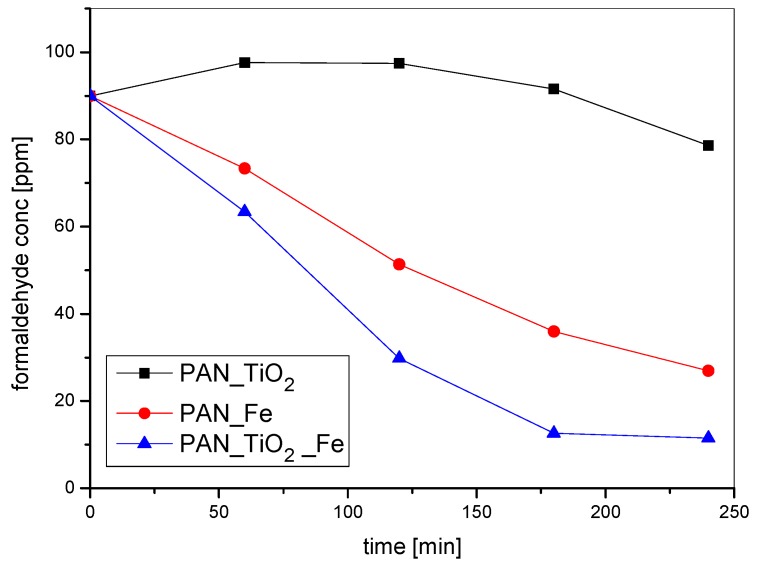
Formaldehyde concentration profile using heterogeneous photo-Fenton (PAN_Fe), photocatalytic (PAN_TiO_2_) and their combination (PAN_TiO_2__Fe) catalysts. The maximum standard deviation of the experimental points was 1.9 ppm.

Figure 8 reports the results when using a heterogeneous system based on photo-Fenton (PAN_Fe), photocatalysis (PAN_TiO_2_) and their combination (PAN_TiO_2__Fe), under UV illumination, pH = 3, without the use of any added H_2_O_2_. It can be noted that the combination of TiO_2_ and Fe^2+^ showed the best result, i.e., there was a synergy deriving from the combination of photocatalysis and photo-Fenton since the combined process gave better results than the simple sum of the two distinct processes.

This synergy can be due to the fact that in presence of iron sulfate the UV absorbance of the system around 290 nm increased, owing to the absorption properties of this compound, therefore favoring UV activation [23]. Moreover, this synergy can be related to the roles of iron as an electron acceptor, to facilitate charge separation in TiO_2_ photocatalyst, thereby reducing the non-desired electron/hole recombination and thus increasing the rate of OH• formation through the first and second reaction in Scheme 1. Similar results have been reported for homogeneous TiO_2_-Fe system in phenol degradation [19], for Azo dye [25] and for bisphenol A [15] and for Fe doped TiO_2_ [20,21,23].

It was also reported that in this system TiO_2_ is beneficial to the fast conversion of Fe^3+^→Fe^2+^ [15], thus favoring the closing the Fe^3+^/Fe^2+^ cycle of reactions mentioned before when dealing with photo-Fenton. Also, the accumulated electrons in the conduction band of TiO_2_ are transferred to oxygen (coming from the air in contact with formaldehyde solution) on the TiO_2_ surface for the formation of O^2−^ or O_2_^2−^, which combines with H^+^ to form H_2_O_2_ [28]. The evolved H_2_O_2_ with FeSO_4_ formed Fenton reagent which can further degrade formaldehyde [28].

Summarizing, the presence of iron was beneficial in promoting the photocatalytic activity of TiO_2_ while TiO_2_ was beneficial in promoting the photo-Fenton reaction.

**Figure 9:**
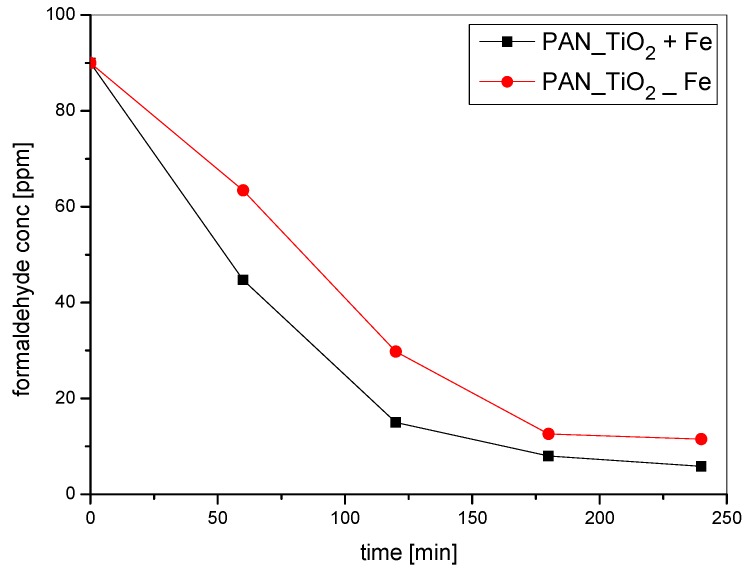
Formaldehyde concentration profile using heterogeneous photo-catalytic-Fenton with Fe supported (PAN_TiO_2__Fe) or dispersed in solution (PAN_TiO_2_+Fe). The maximum standard deviation of the experimental points was 1.8 ppm.

If we compare the results obtained with a fully heterogeneous system (PAN_TiO_2__Fe, where Fe is deposited onto the support) and partly homogeneous one (PAN_TiO_2_+Fe, where 24 ppm of ferrous sulfate was in the aqueous solution, as before in photo-Fenton trials) (Figure 9), it can be noted that the degradation rate was slightly faster for the latter, probably owing to a higher photo-Fenton reaction rate; however, both systems reached a final CH_2_O concentration of around 10 ppm.

**Figure 10:**
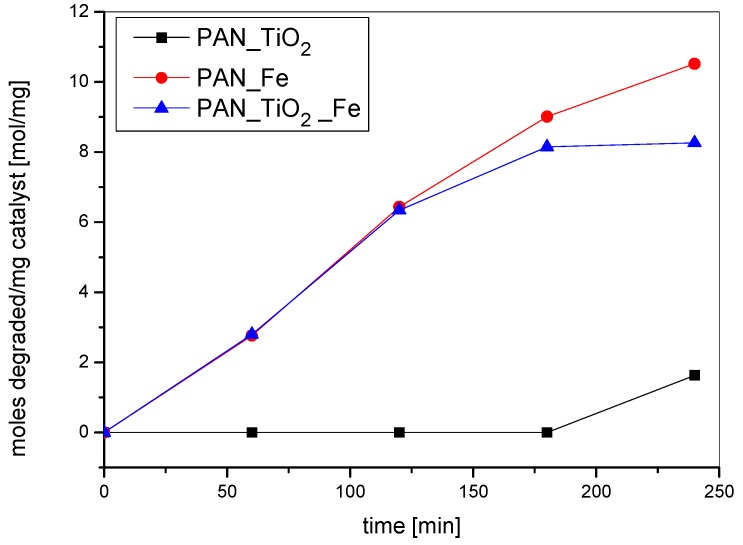
Formaldehyde degraded per mg of catalyst for heterogeneous photo-Fenton (PAN_Fe), photocatalytic (PAN_TiO_2_) and their combination (PAN_TiO_2__Fe) catalysts.

However, if we compare the results on a catalyst weight basis, i.e., considering the moles of formaldehyde degraded per mg of catalyst, it can be shown (Figure 10) that up to 3 h there were no significant differences for PAN_Fe and PAN_TiO_2__Fe; however, over a longer period the best performing system was PAN_Fe. Obviously, this was an initial work and further studies on the optimal iron content need to be carried out.

## 4. Conclusions

Photocatalytic and photo-Fenton processes as well as their combination were studied to degrade formaldehyde aqueous solution; both homogeneous and heterogeneous catalysts were used.

The results showed that the combination of AOPs gave a synergy since the presence of iron was beneficial in promoting the photocatalytic activity of TiO_2_ while TiO_2_ was beneficial in promoting the photo-Fenton reaction.

Moreover, very good results have been obtained using fully heterogeneous nanostructured catalysts (based on TiO_2_ and FeSO_4_) without the need to add H_2_O_2_. In this way, a more economic formaldehyde treatment process has been developed.

## Figures and Tables

**Table 1:** Process parameters for the membranes production.

Materials	Flow Rate (mL/h)	Voltage (kV)	Electrode Distance (cm)	Relative Humidity (%)	Deposition Time (min)
PAN support	2	15 ÷ 20	20 ÷ 25	30 ÷ 40	120
Catalysts deposition	2	23 ÷ 25	15	20 ÷ 30	60

**Table 2:** Catalyst content of the membranes.

Membrane	Catalyst Content (mg/cm^2^)
PAN_TiO_2_	0.14
PAN_Fe	0.12
PAN_TiO_2__Fe	0.19
	(53% TiO_2_ + 47% FeSO_4_)
